# Cholecystectomy in Sweden 2000 – 2003: a nationwide study on procedures, patient characteristics, and mortality

**DOI:** 10.1186/1471-230X-7-35

**Published:** 2007-08-17

**Authors:** Mats Rosenmüller, Markku M Haapamäki, Pär Nordin, Hans Stenlund, Erik Nilsson

**Affiliations:** 1 Department of Surgery, Umeå University Hospital, Sweden; 2 Department of Surgery Östersunds Hospital, Östersund, Sweden; 3 Epidemiology and Public Health Sciences, Umeå International School of Public Health, Sweden

## Abstract

**Background:**

Epidemiological data on characteristics of patients undergoing open or laparoscopic cholecystectomy are limited. In this register study we examined characteristics and mortality of patients who underwent cholecystectomy during hospital stay in Sweden 2000 – 2003.

**Methods:**

Hospital discharge and death certificate data were linked for all patients undergoing cholecystectomy in Sweden from January 1^st ^2000 through December 31^st ^2003. Mortality risk was calculated as standardised mortality ratio (SMR) i.e. observed over expected deaths considering age and gender of the background population.

**Results:**

During the four years of the study 43072 patients underwent cholecystectomy for benign biliary disease, 31144 (72%) using a laparoscopic technique and 11928 patients (28%) an open procedure (including conversion from laparoscopy). Patients with open cholecystectomy were older than patients with laparoscopic cholecystectomy (59 vs 49 years, p < 0.001), they were more likely to have been admitted to hospital during the year preceding cholecystectomy, and they had more frequently been admitted acutely for cholecystectomy (57% Vs 21%, p < 0.001). The proportion of women was lower in the open cholecystectomy group compared to the laparoscopic group (57% vs 73%, p < 0.001). Hospital stay was 7.9 (8.9) days, mean (SD), for patients with open cholecystectomy and 2.6 (3.3) days for patients with laparoscopic cholecystectomy, p < 0.001. SMR within 90 days of index admission was 3.89 (3.41–4.41) (mean and 95% CI), for patients with open cholecystectomy and 0.73 (0.52–1.01) for patients with laparoscopic cholecystectomy. During this period biliary disease accounted for one third of all deaths in both groups. From 91 to 365 days after index admission, SMR for patients in the open group was 1.01 (0.87–1.16) and for patients in the laparoscopic group 0.56 (0.44–0.69).

**Conclusion:**

Laparoscopic cholecystectomy is performed on patients having a lower mortality risk than the general Swedish population. Patients with open cholecystectomy are more sick than patients with laparoscopic cholecystectomy, and they have a mortality risk within 90 days of admission for cholecystectomy, which is four times that of the general population. Further efforts to reduce surgical trauma in open biliary surgery are motivated.

## Background

Gallstone disease is the most common of all abdominal diseases for which patients are admitted to hospital in developed countries [1] and an increase in hospital admission rates was registered in the 1990s [2] The prevalence of gallstone increases with age [3]. Hence, the safety and cost-effectiveness of treatment of gallstone disease in populations with increasing age is of great importance to public health. Cholecystectomy is the treatment of choice. The common bile duct may be cleared of gallstones at cholecystectomy or through endoscopic sphincterotomy, which involves an extra procedure [4].

Length of hospital stay for cholecystectomy became shorter during the 1980s[5,6]. and it was demonstrated that open cholecystectomy could be performed through a smaller incision than traditionally used [7]. Small-incision or mini-laparotomy cholecystectomy was found compatible with ambulatory surgery (3 – 10 hrs postoperative hospital stay) [8]. However, laparoscopic cholecystectomy rapidly became the preferred technique in the early 1990s [9]. Population-based studies demonstrated great variations in cholecystectomy rates between countries before the introduction of the laparoscopic technique [5]. Thereafter, cholecystectomy rates increased in Scandinavia [10] Scotland [6], UK [3] and in the US [11]. At the end of the 1990s 70–80% of all cholecystectomies were completed as laparoscopic procedures [6,12,13]., which then had also been found compatible with ambulatory surgery [14,15]. The shift from open to laparoscopic cholecystectomy has been accompanied by an increased incidence of iatrogenic bile duct injury [16] and an increase in the overall incidence of intra-operative injury[17].

If gallbladder surgery is to be improved we need information on patients undergoing cholecystectomy in defined populations. We have utilised data from nation-wide registers to characterise patients having open or laparoscopic cholecystectomy in Sweden, and studied mortality over the first year after admission for cholecystectomy.

## Methods

Sweden (9.0 million inhabitants) has a public health-care system. The Swedish National Board of Health and Welfare's Epidemiology Centre compiles data on individual hospital discharges in the Hospital Discharge Register. Since 1987 the register has included all Swedish hospitals [18]. The record of each hospital stay contains diagnoses at discharge coded according to the Swedish version of the International Classification of Diseases, from 1997 10th revision (ICD10). Surgical procedures are classified according to the Swedish version of Classification of Surgical Procedures. All operations are classified as finally completed. This means that laparoscopic cholecystectomies converted to open operations are stated as open procedures in the register database. Underlying causes of death are coded by the ICD10 classification. Patients are identified through a national registration number unique for each resident in Sweden. In the file for each stay it is indicated whether the admission was elective or acute. The exact date of operation during an admission is not recorded in the Hospital Discharge Register. Hence, we have information on *hospital stay *but not on *postoperative stay*. The time at risk for death was calculated as the difference between the date of index admission and the date of death or the end of follow-up (December 31^st ^2004), whichever occurred first. The patients' pre- or post-index admissions were characterised according to their primary diagnosis (biliary disease, heart-lung disease, or any other diagnosis) and by surgical procedures performed during these admissions. Procedures performed before, during, or after index stay are classified as described in the Appendix.

For all records reported to the Hospital Discharge Register a data control is run. A check is made that compulsory variables are reported, e.g. personal identification number, hospital and main diagnosis. A check is also made that codes for different variables and dates have valid values. Some obviously incorrect data are corrected in connection with the quality controls. In 2003 the main diagnosis was missing in 0.9 per cent of the hospital stays reported. For acute somatic care 0.5 per cent was missing.

### Patients

All admissions from January 1^st ^2000 through December 31^st ^2003 with cholecystectomy procedure codes (JKA20, JKA21) were selected from the Hospital Discharge Register. In order to ascertain information concerning hospital admissions one year before and one year after cholecystectomy (index) admission we obtained records for all admissions from January 1^st ^1999 through December 31^st ^2004. The study base initially comprised 44821 patients. After exclusion of patients with malignant or benign intra-abdominal or kidney tumour with a procedure code for tumour resection, 44084 patients remained.

### Statistics

Proportions have been compared using the chi square test or Fisher exact test when appropriate. Location of two groups of ratio scale variables were compared using independent samples t-test or Mann-Whitney U-test. A p-value less than 0.05 was considered significant.

## Results

Of the 44084 patients who underwent cholecystectomy 2000 – 2003, 12675 patients (29%) were treated by open cholecystectomy and 31409 patients (71%) by laparoscopic cholecystectomy, see Table 1. Acute or chronic gallbladder disease (calculous or acalculous) was the indication for cholecystectomy in 41772 patients giving an incidence of 116 per 100 000 inhabitants per year. Disease in the bile ducts accounted for cholecystectomy in 305 patients and acute pancreatitis in 995 patients. The group classified as "other diagnoses" comprised patients with a variety of malignant or benign diagnoses including malignancy of the upper GI-tract without tumour resection, as well as diagnoses unrelated to gallbladder disease such as appendicitis, gastritis, or ileus. Patients in this group were excluded from further analysis.

**Table 1:** Primary diagnosis at index (cholecystectomy) stay, n = 44084

	Open cholecystectomy n = 12675		Laparoscopic cholecystectomy n = 31409		p-value*
	n	%	n	%	
Gallbladder disease	11363	89.6	30409	96.8	
Bile duct disease	192	1.5	113	0.4	
Acute pancreatitis	373	2.9	622	2.0	
Other diagnoses	747	5.9	265	0.8	
					< 0.001

*calculated with the chi-square test

Table 2 illustrates age, gender, and mode of admission (acute/elective), and length of index (cholecystectomy) stay for patients who underwent cholecystectomy for biliary disease including acute pancreatitis 2000–2003. Mean age of patients was 59.2 year in the open group and 48.7 year in the laparoscopic group, and the percentage of women was 56.5% and 73.1%, respectively. In the open group 56.6% of all patients had an acute admission compared to 20.9% in the laparoscopic group (p < 0.001 for all analyses). Hospital stay for all patients was 4.1 (5.9) days, mean (SD). It was significantly longer for patients in the open cholecystectomy group, 7.9 (8.9) vs 2.6 (3.3) days, p < 0.001.

**Table 2:** Age and gender of patients; mode of admission; and length of index (cholecystectomy) stay, n = 43072

	Open cholecystectomy n = 11928	Laparoscopic cholecystectomy n = 31144	p-values *
Age, mean (SD)	59.2 (17.1)	48.7 (15.5)	< 0.001
Women (%)	56.5	73.1	< 0.001
Acute admission No %	56.6	20.9	< 0.001
Length of stay mean, (SD)	7.9 (8.9)	2.6 (3.3)	< 0.001

* calculated using chi-square test and t-test.

During the year preceding index admission, a total of 6046 of 11928 patients (50.7%) in the open group and 12456 of 31144 patients (40.0%) in the laparoscopic group were admitted to hospital, p < 0.001. Length of stay for heart-lung disease, for biliary disease, and for all other diagnoses was longer for patients in the open compared to patients in the laparoscopic group, see Table 3. Patients in the open group were also more likely to have undergone at least one sphincterotomy prior to cholecystectomy admission (Table 4).

**Table 3:** Hospital admission and length of stay during one year prior to index (cholecystectomy) stay, n = 43072.

	Open cholecystectomy n = 11928			Laparoscopic cholecystectomy n = 31144			
Admissions†, main diagnosis	Admissions n	Days Mean	Days (SD)	Admissions n	Days mean	Days (SD)	p-values

Heart-lung disease	1451	10.5	(16.2)	1615	6.2	(9.2)	< 0.001
Gallbladder/bile duct disease	3721	6.2	(7.6)	8264	4.0	(4.5)	< 0.001
Other diagnoses	2427	7.9	(16.1)	4883	5.1	(12.8)	< 0.001

†one patient may have several admissions with different and/or same diagnoses.

**Table 4:** Interventions during one year prior to index (cholecystectomy) stay, no = 43072.

**Interventions**	Open cholecystectomy n = 11928		Laparoscopic cholecystectomy n = 31144		
Procedure	Patients	%	Patients	%	p-values

Sphincterotomy	510*	4.3	782**	2.5	< 0.001
Other open procedures	36	0.3	22	0.1	< 0.001
Other laparoscopic procedures	4	0.0	2	0.0	0.094
Other endoscopic procedures	414	3.5	497	1.6	< 0.001
Percutaneous procedures	15	0.1	3	0.0	< 0.001

* including 20 patients with sphincterotomy more than once

** including 15 patients with sphincterotomy more than once

Table 5 shows surgical procedures performed in addition to cholecystectomy during the index stay. Intra-operative cholangiography was performed on 72% of patients in both groups. The common bile duct was explored in 17.1% of patients in the open cholecystectomy group, but only 0.8% in the laparoscopic group, p < 0.001. Laparoscopic removal of common bile duct stones through the cystic duct was used in 1.0% of patients in the open group, suggesting that these patients had undergone converted laparoscopic procedures. 1.1% of patients in the open cholecystectomy group, but only 7 (0.06%) patients in the laparoscopic group, p < 0.001, underwent bilio-digestive anastomosis (creation of a shunt between the bile tree and the gastrointestinal tract). Other additional surgical procedures were also more frequent in the open cholecystectomy group.

**Table 5:** Concomitant interventions during index (cholecystectomy) stay, n = 43072.

	Open cholecystectomy n = 11928		Laparoscopic cholecystectomy n = 31144		p-value
Procedure	n	%	n	%	

Intra-operative cholangiography	8534	71.5	22486	72.2	0.176
Intra-operative cholangioscopy	1077	9.0	505	1.6	< 0.001
Common bile duct exploration	2035	17.1	262	0.8	< 0.001
Sphincterotomy	345	2.9	695	2.2	< 0.001
Laparoscopic stone extraction via cystic duct	114	1.0	581	1.9	< 0.001
Bilio-enteric anastomosis without resection	126	1.1	7	0.0	< 0.001
Excision of bile duct	23	0.2	0	0.0	< 0.001
Other operations on bile duct	20	0.2	12	0.0	< 0.001
Re-intervention on bile duct	15	0.1	4	0.0	< 0.001
Vessel suture	11	0.1	0	0.0	< 0.001
Bowel suture	34	0.3	6	0.0	< 0.001
Other open procedures	273	2.3	130	0.4	< 0.001
Other laparoscopic procedures	105	0.9	79	0.3	< 0.001
Other endoscopic procedures	322	2.7	579	1.9	< 0.001
Percutaneous procedures	25	0.2	14	0.0	< 0.001

Table 6 illustrates readmissions and length of stay per admission for biliary disease, for heart-lung disease, and for any other cause within one year of index stay. In the open group 3447 (29%) patients were readmitted and in the laparoscopic group 5151 patients (17%), p < 0.001. The length of stay was longer for the open group regardless of main diagnosis. During readmission there were no significant differences between the open and the laparoscopic group with respect to sphincterotomy or rare procedures such as excision of bile duct, whereas bilio-enteric anastomosis, operation for incisional hernia, and percutaneous procedures were more frequent in the open cholecystectomy group, see Table 7.

**Table 6:** Readmission and hospital stay within one year of index (cholecystectomy) stay, n = 43072

	Open cholecystectomy n = 11928			Laparoscopic cholecystectomy n = 31144			p-values
Readmission†	Admissions No	Days Mean	Days (SD)	Admissions No	Days Mean	Days (SD)	

Biliary disease	748	9.5	(17.0)	848	5.8	(10.2)	< 0.001
Heart-lung disease	1158	14.3	(23.4)	1214	8.6	(13.4)	< 0.001
Other diagnoses	2246	10.6	(25.6)	3699	7.9	(26.2)	< 0.001

†one patient may have several admissions with different and/or same diagnoses

**Table 7:** Interventions during readmission within one year of index (cholecystectomy) stay, n = 43072

	Open cholecystectomy n = 11928		Laparoscopic cholecystectomy n = 31144		p-values
Intervention	n*	%	n*	%	

Sphincterotomy	163	1.4	286	0.9	0.360
Excision of bile duct	8	0.0	9	0.0	0.603
Bilio-enteric anastomosis without resection	18	0.2	11	0.0	0.016
Other open procedures	122	1.0	75	0.2	<0.001
Other operations on bile duct	5	0.0	6	0.0	0.716
Incisional hernia	97	0.8	52	0.2	< 0.001
Other laparoscopic procedures	1	.0	1	0.0	-
Other endoscopic procedures	201	1.7	293	0.9	0.564
Percutaneous procedures	25	0.2	5	0.0	< 0.001

* number of interventions in respective group

% per cent of patients in respective group

From Tables 4, 5, and 7 it may be deduced that 8.6% of open cholecystectomies were associated with sphincterotomy at least once as compared to 5.6% of laparoscopic cholecystectomies. Furthermore, common bile duct exploration or transcystic stone extraction was done on 18.1% of patients in the open group and 2.7% in the laparoscopic group. Of all patients undergoing cholecystectomy, 1292 patients were admitted to hospital for sphincterotomy before the index stay and 449 after the index stay.

Table 8 shows the number of deaths and standardised mortality ratio, SMR, for patients who died within 90 days, and between 91 and 365 days after index admission. The mortality of patients who had an open procedure was increased fourfold compared to the mortality of the corresponding general population over the three-month period after index admission. During the following nine months mortality was significantly higher than expected for patients younger than 69 years. However, for all patients in the open group SMR was 1.01 (0.87–1.16). For patients in the laparoscopic group, SMR within 90 days was 0.73 (0.52–1.01) and between 91 and 365 days 0.56 (0.44–0.69).

**Table 8:** Deaths and standardised mortality ratio (SMR)

**Open cholecystectomy**							
Days after index admission		0–90			91–365		
Age, years	Patients	Deaths	SMR	CI 95%	Deaths	SMR	CI 95%

0–64	6823	38	6.84	4.84–9.39	39	2.34	1.66–3.20
65–69	1230	17	3.95	2.30–6.32	22	1.70	1.07–2.58
70–79	2539	69	3.43	2.67–4.35	56	0.93	0.70–1.21
≥80	1336	114	3.65	3.01–4.38	68	0.72	0.56–0.92
Total		238	3.89	3.41–4.41	185	1.01	0.87–1.16

							
**Laparoscopic cholecystectomy**							
							
Days after index admission		0–90			91–365		

Age, years	Patients	Deaths	SMR	CI 95%	Deaths	SMR	CI 95%

0–64	25822	7	0.46	0.18–0.94	31	0.67	0.46–0.96
65–69	2068	4	0.61	0-17-1.57	16	0.82	0.47–1.33
70–79	2712	17	0.91	0.53–1.46	26	0.47	0.30–0.68
≥80	542	9	0.90	0.41–1.71	11	0.37	0.18–0.66
Total		37	0.73	0.52–1.01	84	0.56	0.44–0.69

The causes of death for all patients who died within one year of cholecystectomy are shown in Table 9. In both the open and the laparoscopic group, approximately one third of all deaths within 90 days of admission could be ascribed to biliary disease. During the subsequent nine months biliary disease was a rare cause of death (6 of 185 deaths in the open group, zero of 84 deaths in the laparoscopic group).

**Table 9:** Cause of death.

0–90 days after index admission		
Cause of death	Open cholecystectomy n = 11928	Laparoscopic cholecystectomy n = 31144

Gallbladder disease	74	9
Biliary tree disease	9	2
Acute pancreatitis	9	0
Heart-lung disease	64	13
Other disease	82	13
Total	238	37

91 – 365 days after index admission		

Cause of death	Open cholecystectomy	Laparoscopic cholecystectomy

Gallbladder disease	2	0
Biliary tree disease	2	0
Acute pancreatitis	2	0
Heart-lung disease	43	27
Other diagnoses	136	57
Total	185	84

## Discussion

A decade after the introduction of laparoscopic cholecystectomy, 28% of all patients with benign biliary disease had their cholecystectomy completed as an open procedure. Patients in the open group were more likely to have an acute admission for cholecystectomy, they were ten years older than patients in the laparoscopic group, and they were more likely to have complications of gallstone disease (acute pancreatitis and common bile duct stones). They had spent more time in hospital before index admission, indicating that they were more fragile than patients in the laparoscopic group. Hospital stay at cholecystectomy was longer in the open compared to the laparoscopic cholecystectomy group. Mortality of patients in the open group was increased fourfold within three months of index admission, but close to that of the corresponding general population during the following nine months. Patients having laparoscopic cholecystectomy had a reduced mortality over the year following index admission, indicating that they were healthier than the Swedish population in general. In both cholecystectomy groups, one third of all deaths within 90 days of index admission were due to biliary causes.

Information was retrieved from a database covering all patients having inpatient cholecystectomy in Sweden 2000–2003. We lack information on cholecystectomy done as a day-case procedure, estimated to be some 13% of all cholecystectomies performed in Sweden during 2002 [19]. In population-based studies such as the present one, conversion to open cholecystectomy comprises some 10% of all laparoscopic cholecystectomies [12,20]. These are classified as open cholecystectomies making it impossible to study the relative merits of open and laparoscopic cholecystectomy on an intention to treat basis. However, our study raises questions concerning cholecystectomy incidence, selection of patients for laparoscopic versus open cholecystectomy, and technique used for open cholecystectomy.

We observed an incidence of 116 per 100000 inhabitants per year for gallbladder disease, demonstrating that cholecystectomy rate in Sweden has prevailed at a significantly higher level than before the introduction of the laparoscopic technique [13]. There is no consensus as to which or how many patients with gallstone disease should be offered cholecystectomy. The proportion of gallstone patients having cholecystectomy varies in defined populations from 5% to 55% [5]. On the basis of a recent randomised controlled trial [21] it was concluded that watchful waiting may be a safe option for patients with symptomatic non-complicated gallstone disease. It has been shown that the prevalence of abdominal pain is significantly higher in cholecystectomised subjects than in subjects with a normal gallbladder [22,23]. Obviously, there is a need for further studies to gain firm evidence on which to base advice to patients with gallstones and abdominal symptoms concerning cholecystectomy.

Patients who had open cholecystectomy were older, had a higher rate of emergency admission, and a higher co-morbidity than patients with laparoscopic cholecystectomy. From three months to one year after admission for cholecystectomy standardised mortality ratio for patients in the open cholecystectomy group did not differ from that in the general population, whereas patients with laparoscopic cholecystectomy had a significantly reduced standardised mortality ration, indicating that they represent individuals which are healthier than the general population in Sweden. Within three months after admission for cholecystectomy standardised mortality ratio was increased fourfold compared to that of the general population in the open group but lower than expected for patients in the laparoscopic group. The most striking difference in standardised mortality ratio within three months after admission for cholecystectomy was seen for patients younger than 65 years, 6.84 (4.84 – 9.39) for open cholecystectomy and 0.46 (018 – 0.94) for laparoscopic cholecystectomy. It is not surprising that our patients with open cholecystectomy had a high standardised mortality ratio as high age, acute admission, and hospital admission within three months prior to cholecystectomy have been associated with increased mortality[24]. We found that one third of all patients who died within 90 days of cholecystectomy had biliary disease as the underlying cause of death, whereas this was unlikely for the rest of the postoperative year. This concurs with a study demonstrating an increased mortality up to 90 days after cholecystectomy [6] but not thereafter.

Use of cholangiography is to be recommended as it has been found to reduce the risk for common bile duct injury [25], and in the present audit intraoperative cholangiography was performed in 72% of all cholecystectomies with no difference between the open and the laparoscopic group. We found an overall incidence of endoscopic sphincterotomy (before, during, and after index admission) of 8.6% in the open cholecystectomy group compared to 5.6% in the laparoscopic group. When common bile duct exploration was indicated during cholecystectomy, it was preferably done at open cholecystectomy, 17.1% in open cholecystectomy vs 0.8% for laparoscopic cholecystectomy. An increase in endoscopic sphincterotomy in the 1990s has been noted in England [2] and in Germany[26], paralleled by a decline in common bile duct exploration. However, open bile duct surgery will always be required in a proportion of patients with common bile duct stones. The decline in training in open biliary surgery has affected junior surgeons' attitude to bile duct surgery [27], and it has increased the risk for technical complications during bile duct exploration[28]. This is a matter of concern as rare but serious complications following endoscopic sphincterotomy are well-known [29]. In a long-term follow-up after endoscopic sphincterotomy, early complications were seen in 16% of patients and late complications in 24% of patients [30].

In view of the excess mortality in open cholecystectomy seen in this study it is appropriate to consider the use of small-incision cholecystectomy, which according to a Cochrane review [31] is preferable to open cholecystectomy if expertise is available. Systematic reviews have also shown that laparoscopic cholecystectomy and small-incision cholecystectomy should be regarded as equal with respect to postoperative recovery and complication, although small-incision cholecystectomy is associated with a shorter operating time [5,32]. Further, the long-term cosmetic effects of laparoscopic and small-incision open cholecystectomy do not differ significantly [33]. However, data from a Swedish national quality register [34] for gallbladder surgery indicate that small-incision cholecystectomy was used for less than five per cent of cholecystectomies in Sweden 2006.

## Conclusion

We have demonstrated that open cholecystectomy, the most traumatic of cholecystectomy methods used today, has been preferred for our most vulnerable patients, either as primary procedure or as conversion from laparoscopic cholecystectomy. There is an obvious need for further studies of techniques used in gallbladder surgery. This is of particular importance in populations with increasing age [35]. Future studies in this field have to recognise the substantial learning curve necessary to master the "video-eye-hand" coordination involved in the laparoscopic technique[17] and they should have a cost-utility approach.

## Competing interests

The author(s) declare that they have no competing interests.

## Authors' contributions

MR, MH, PN, HS, and EN designed the study; HS performed the statistical analyses; EN drafted the manuscript. All authors have contributed to, read, and approved the final version of the manuscript.

## Appendix

See Table 10

**Table:** Operations classified for the present study

*Cholecystectomy-related procedures (JKA20, JKA21 excluded)*	
Intraoperative cholangiography	TJK00, TJK01
Intraoperativ cholangioscopy	JKB20, TJK21
Common bile duct exploration	JKB00, JKB01
Laparoscopic transcystic stone removal	JKB11
Bilio-enteric anastomosis without resection	JKD
Endoscopic sphincterotomy	JKE02
Excision of bile duct	JKC
Reintervention on bile duct	JKF
Vessel suture	PCB, PCC
Bowel suture	JFA70, JFA71
Reoperations due to complications after previous abdominal surgery	JW
	
*Other open procedures*	
Cholecystotomy	JKA00
Cholecystostomy	JKA10
Other operation on gallbladder	JKA96
Bile duct suture	JKB40
Other local operation on bile duct	JKB96
Transduodenal sphincterotomy	JKE00
Transduodenal sphincteroplasty	JKE06
Other transduodenal procedure on bile duct or sphincter	JKE96
Other operation on bile duct	JKW96
Incisional hernia repair	JAD
	
*Other laparoscopic procedures*	
Laparoscopic cholecystostomy	JKA11
Other laparoscopic procedure on gallbladder	JKA97
Other laparoscopic local operation on bile duct	JKB97
Other laparoscopic operation on bile duct	JKW97
	
*Other endoscopic procedures*	
Removal of common bile duct stones	JKE12
Lithotripsy	JKE15
Internal drainage	JKE18
Removal of foreign body (stent)	JKE22
External drainage	JKE25
Dilatation of bile duct	JKE32
Other endoscopic procedure	JKE98, JKW98
	
*Percutaneous procedures*	
Cholecystostomy	JKA13
Transhepatic drainage	JKB30

## Pre-publication history

The pre-publication history for this paper can be accessed here:


